# Supplementary material for: Ovaries absent links dLsd1 to HP1a for local H3K4 demethylation required for heterochromatic gene silencing
Source: eLife. 2019 Jan 16;8:e40806. doi: 10.7554/eLife.40806 (PMC6335052; doi:10.7554/eLife.40806)
Supplement: Supplementary file 1. [file elife-40806-supp1.docx]

**Supplementary file 1**

| Offspring of *ova^1^/ CyO sibling crosses* | | Number | Ratio (%) | Expected % based on Mendelian inheritance |
| --- | --- | --- | --- | --- |
| *ova^1^/ CyO* | male | 438 | 47.3 | 33.3 |
|  | female | 418 | 45.2 | 33.3 |
| *ova^1^/ ova^1^* | male | 1 | 0.1 | 16.7 |
|  | female | 68 | 7.4 | 16.7 |
| Offspring of *ova^1^/ CyO and ova^4^/CyO sibling crosses* | | Number | Ratio (%) | Expected % based on Mendelian inheritance |
| *ova^1^/CyO or ova^4^/CyO* | male | 213 | 44.7 | 33.3 |
|  | female | 186 | 39.0 | 33.3 |
| *ova^1^/ova^4^* | male | 2 | 0.4 | 16.7 |
|  | female | 76 | 15.9 | 16.7 |

**Supplementary table 1.** Viability test of *ova* mutants.

**Supplementary table 2.** Primers used in the study.

| Primer Sequence | |
| --- | --- |
| Het-A_forward | CGCGCGGAACCCATCTTCAGA |
| Het-A_reverse | CGCCGCAGTCGTTTGGTGAGT |
| TAHRE_forward | CTGTTGCACAAAGCCAAGAA |
| TAHRE_reverse | GTTGGTAATGTTCGCGTCCT |
| ova_forward | ACAACTGTGATGCCGAACTT |
| ova_reverse | TCCGTTGATGTTTCTTCGCT |
| GFP_forward | TGCTTCAGCCGCTACCCCGA |
| GFP_reverse | AACTTCACCTCGGCGCGGGT |
| actin5c_forward | GTTGCTGCTCTGGTTGTCG |
| actin5c_reverse | CACACGCAGCTCATTGTAG |
| burdock_forward | AGGGAAATATTTGGCCATCC |
| burdock_reverse | TTTTGGCCCTGTAAACCTTG |
| blood-RA_forward | CCAACAAAGAGGCAAGACCG |
| blood-RB_forward | TCGAGCTGCTTACGCATACTGTC |
| mdg1_forward | AACAGAAACGCCAGCAACAGC |
| mdg1_reverse | CGTTCCCATGTCCGTTGTGAT |
| Tart_forward | AGAGAGGGAAAGAAGGGAAAGGGA |
| Tart_reverse | ATTTCCTGCCTGGTTAGATCGCCA |
| Gypsy_forward | CTTCACGTTCTGCGAGCGGTCT |
| Gypsy_reverse | CGCTCGAAGGTTACCAGGTAGGTTC |
|  |  |
